# Retrospective investigation of retinoblastoma in Chinese patients

**DOI:** 10.18632/oncotarget.18174

**Published:** 2017-05-23

**Authors:** Liwen Jin, Wei Zhang, Hong Pan, Tengyan Li, Beihong Liu, Junyang Zhao, Binbin Wang

**Affiliations:** ^1^ Department of Ophthalmology, Maternal and Children’s Hospital, Quanzhou, Fujian, China; ^2^ Center for Genetics, National Research Institute for Family Planning, Beijing, China; ^3^ Graduate School of Peking Union Medical College, Beijing, China; ^4^ Department of Ophthalmology, Beijing Children's Hospital, Capital Medical University, Beijing, China

**Keywords:** retinoblastoma, retrospective investigation, enucleation, prognosis, retina

## Abstract

This is a retrospective investigation of patients with Retinoblastoma (RB) conducted from 2013 to 2016 at the Quanzhou Maternal and Child Health Hospital (China). Demographic and clinical characteristics, treatment outcomes, and risk factors were studied.

In total, 436 patients were included in the study. Most of the findings obtained in this study are consistent with other previous reports. The male: female ratio was 1.41:1, and the unilateral: bilateral ratio was 1.51:1. Leukocoria was the most common presenting sign (79.44%), followed by strabismus (12.38%). While, the overall rates of enucleation (15.82%) and mortality (0.92%) were markedly lower than in other reports of RB in Chinese, and most of the patients received conservative therapy. There were signficant differences (*p* < 0.001) in the age of at first sign and diagnosis, and treatment modalities between patients with bilateral and unilateral RB. The treatment modalities did not show a specific trend over the 3-year study period. Our results suggest that an incorrect initial diagnosis and long lag time may be risk factors for ineffective treatment and a poor prognosis in patients with RB.

This was a comprehensive retrospective investigation in which the sample size exceeded most previous retrospective investigations of RB. Our study confirmed that early detection, accurate diagnosis, and active intervention are conducive to control of retention of patients’ vision. Fundus examinations, education regarding the early signs of RB, and optimization of the therapeutic strategy of RB may play important roles in ocular health.

## INTRODUCTION

Retinoblastoma (RB) is the most common primary intraocular malignancy of infancy and childhood, and seriously endangers the vision and life of children [1]. Untreated, its mortality rate is 100%. The exact pathogenesis of RB remains unclear. It is clearly heterogeneous, with a wide spectrum of causes including genetic alterations (*RB1* mutation), infectious diseases, and somatic mutations [2]. The worldwide incidence of RB is approximately 1 per 15,000 to 20,000 live births, which corresponds to 7,000 to 8,000 new cases every year [3]. RB reportedly has no validated geographic or population hotspots, but it is associated with significant differences in visual impairment and mortality in different countries or regions [1]. Modern technology and good medical access have decreased the RB-associated mortality rates in Europe and the US to about 5% and 3%, respectively [3]. However, the greatest burden is present in large populations with high birth rates, such as Africa and Asia. The RB-associated mortality rates in Africa and Asia (excluding Japan) are 70% and 39%, respectively [3].

China is the most populous country and documents about 1,000 new cases of RB per year, accounting for 1/8 of all cases worldwide [4]. Although the level of medical care in China has significantly improved, the survival rate of children with RB remains lower and the enucleation rate remains higher than many developed countries [1]. Survival and the chance of saving vision depend on the severity of disease at presentation. Early screening, detection, diagnosis, and intervention play a crucial role in the prevention and reduction of blindness and vision loss caused by ocular disorders.

To better understand the clinical characteristics, diagnosis, treatment, and prognosis of RB in China, we performed a retrospective observational investigation on the clinical data of 436 patients at Quanzhou Maternal and Child Health Hospital (Fujian, China) from 2013 to 2016.

## RESULTS

### Demographic features

The demographic features of the 436 patients with RB are listed in Table 1. The male: female ratio was 1.41:1. The RB occurred unilaterally in 60.09% patients and bilaterally in 39.91% patients. Of the 610 affected eyes, 47.70% were left eyes and 52.30% were right eyes. The median age of the patients was 27 months (range, 14 days to 408 months). The median age at which the first sign was noted by the parents or family members (onset age) was 17 months (range, birth to 384 months). The median age at diagnosis was 18 months (range, 1–390 months). The median lag time was 1 month (range, 1 day to 96 months).

**Table 1:** General characteristics of 436 cases with retinoblastoma

Gender	
Male	255 (58.49%)
Female	181 (41.51%)
Laterality	
Unilateral	262 (60.09%)
vBilateral	174 (39.91%)
Affected eye (*n* = 610)	
Right eye	319 (52.30%)
Left eye	291 (47.70%)
Median age, mo	27 (range, 0.47–408)
Median onset age, mo	17 (range, 0–389)
Median diagnosis age, mo	18 (range, 1–390)
Median lag time, mo	1 (range, 0.03–96)
Family history of RB	2 (0.46%)

**Figure 1:**
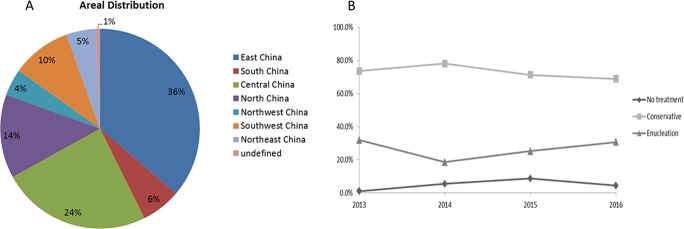
Geographical distribution and treatment of RB patients (**A**) The RB patients in our study were from 31 provincial administrative regions of China. Most patients were from East China (36%) followed by Central China (24%); (**B**) The rate of treatment modalities during 2013 to 2016, there was no specific trends in were observed during the 3-year study period.

The patients were from 31 of 35 provincial administrative regions of China (except Tibet, Hong Kong, Taiwan and Macao). Most patients were from the province where the hospital was located. Patients from East China and Central China accounted for more than half (60%) of the total patients (Figure 1A).

### Symptoms at presentation

Presenting symptoms were reported in only 428 of the 436 patients because of a lack of information. As shown in Table 2, the most frequent symptom at presentation was leukocoria (79.44%). Other presenting symptoms, according to the ratio from high to low in order, were strabismus, red eye, decreased vision, lacrimation, photophobia, pain and proptosis.

**Table 2:** Symptoms at presentation* (*n* = 428)

Leukocoria	340 (79.44%)
Strabismus	53 (12.38%)
Red eye	27 (6.31%)
Decreased vision	16 (3.74%)
Lacrimation	7 (1.64%)
Photophobia	4 (0.93%)
Ocular pain	3 (0.70%)
Proptosis	1 (0.23%)

*Patients presented with one or more symptoms.

### Treatment modalities and outcomes

Of the 434 patients with RB (2 patients’ treatment data were not available; with unilateral RB and the other with bilateral RB), 95 patients (27 unilateral, 68 bilateral) underwent enucleation (21.89%); one of these patients underwent bilateral enucleation. The overall enucleation rate was 15.82% (96/607). A total of 73.96% (321/434) patients received conservative therapy, including pars plana vitrectomy (PPV), chemotherapy, radiation therapy, laser photocoagulation, cryotherapy, and local resection. Notably, 18 (4.1%) patients received no treatment.

Four (0.92%) children died, and all had bilateral RB. All deaths were due to metastasis of RB but not other malignant neoplasms or accidents. No specific trends in treatment modalities were observed during the 3-year study period (Figure 1B). The enucleation rate increased and the conservative treatment rate decreased from 2014. The non-treatment rate increased from 2013 and began to decrease in 2015.

### Comparison of unilateral and bilateral groups

**Figure 2:**
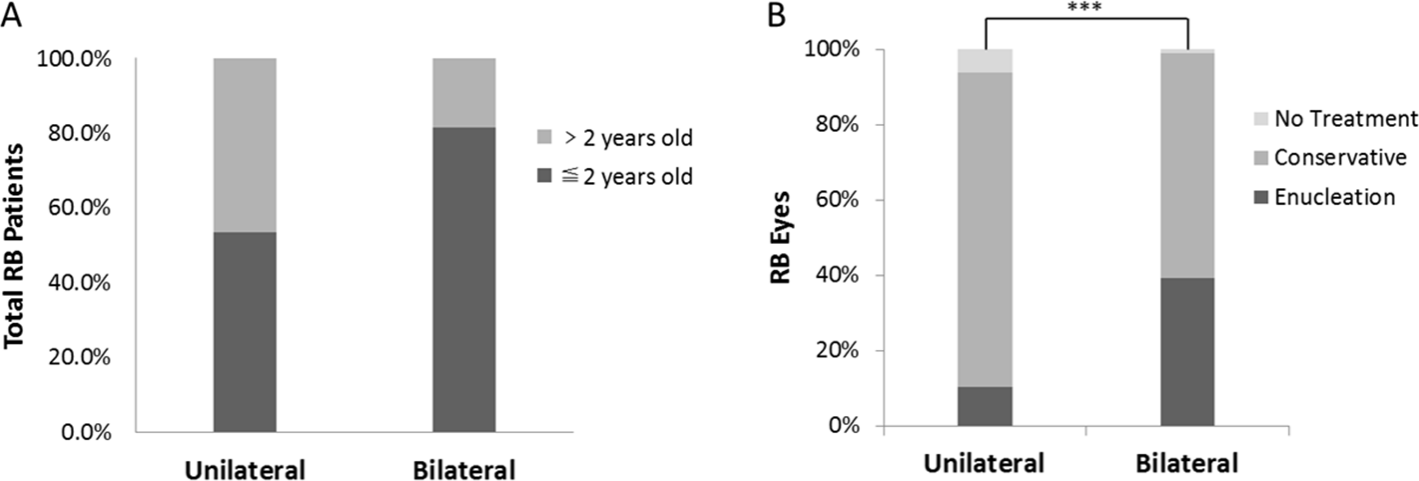
Difference between unilateral and bilateral groups (**A**) The relationship between the age at first sign and laterality of RB. (**B**) There was a significant difference in treatment modalities between unilateral and bilateral RB groups (^***^p < 0.001, chi-square test).

As shown in Table 3, the median age at onsetand at diagnosis was younger in patients with bilateral than unilateral RB (*p* < 0.0001, Mann–Whitney U test). The median lag time and sex distribution were not significantly different between the two groups. A total of 57.9% and 85.5% of patients with unilateral and bilateral RB, respectively, showed the first symptoms before 2 years of age (Figure 2A). A total of 53.5% and 81.5% of patients with unilateral and bilateral RB, respectively, were diagnosed before 2 years of age.

**Table 3:** Comparison of demographic features between unilateral and bilateral groups

	Unilateral	Bilateral	*P*
Median age, mo			
At onset (range)	22 (0–389)	11 (0–74)	< 0.000^1*^
At diagnosis (range)	24 (1–390)	13 (1–75)	< 0.0001^*^
Median lag time (mo, range)	1 (0.03–96)	1 (0.2–12)	0.213^*^
Gender			
Male	154 (58.8%)	101 (58.0%)	﹥ 0.05^#^
Female	108 (41.2%)	73 (42.0%)	

^*^Mann–Whitney *U* test

^#^chi-square test

A significant difference in treatment was observed between patients with unilateral and bilateral RB (*p* < 0.001, chi-square test) (Figure 2B). The enucleation rate was higher in patients with bilateral than unilateral RB (39.3% vs. 10.3%, respectively), while the rates of conservative treatment (59.5% vs. 83.5%, respectively) and no treatment (1.2% vs. 6.1%, respectively) showed opposite results.

### Family history

Of the 436 patients, only 2 (0.46%) had a family history of RB. One was diagnosed with bilateral RB presented with leukocoria at 4 months of age, and then underwent pars plana vitrectomy. Her mother had undergone enucleation because of RB 20 years previously. The other patient was diagnosed with unilateral class A RB according to the International Intraocular Retinoblastoma Classification (IIRC) and underwent laser treatment. His older brother was also diagnosed with RB. Unfortunately, we were unable to obtain more detailed information about his brother. Notably, one patient’s mother underwent enucleation of the right eye because of uveitis at 1 year of age.

### Risk factors

Nine patients (2.06%) in our cohort had an incorrect initial working diagnosis or referral diagnosis. Four patients were initially diagnosed with strabismus, and two were initially diagnosed with conjunctivitis. The other three patients were initially diagnosed with glaucoma, uveitis, and trichiasis, respectively. All of these nine patients received incorrect initial treatment, and four of them had a lag time of > 2 months. Two of these nine patients underwent enucleation; thus, the enucleation rate of these nine patients was slightly higher than the average rate in our study (22.22% vs. 21.89%, respectively). Six patients were treated with pars plana vitrectomy, and one patient was treated with chemotherapy. Of the 95 patients who underwent enucleation, the lag time was < 2 months in 81 patients and >2 months in 10 patients (data on the lag time were unavailable in 4 patients). There was no significant association between the lag time and the enucleation rate between these two groups of patients (15.6% vs. 13.9%, respectively; *p >* 0.5).

## DISCUSSION

Childhood blindness and low vision are both severe public health problems that cause heavy social and economic burdens worldwide [5]. RB is an aggressive eye cancer of infancy and childhood that has been historically associated with a poor visual prognosis [6, 7]. Low awareness of RB in the general public and medical community as well as an absence of rigorous clinical trials have impeded the progress of early detection and treatment of RB. Africa and some areas of Asia, in which the economic and medical infrastructure is relatively poor, have higher RB-associated mortality rates than many developed countries in Europe and Americas [3]. Increasing emphasis on ocular health, especially eye disorders resulting in severe vision loss in children, may help improve the current situation.

In this retrospective analysis, we investigated the demographic and clinical features, treatment, and outcomes of 436 Chinese patients with RB. Many of the findings obtained in this study are consistent with other previous reports [4, 8]. Our study included more male than female patients, and most had unilateral, nonhereditary RB. The most common presenting sign was leukocoria. More than half of all patients were from East China and Central China, including the province in which the hospital is located. The population size, economic and medical conditions, and travel time from the patients’ home to the hospital may have been influencing factors. The median age at diagnosis of the tumors of all patients included in our study (18 months) was markedly younger than in two other reports of Chinese patients with RB (25 and 30 months) [4, 8].

Patients with bilateral RB were significantly younger at onset of the first sign and at the time of diagnosis than patients with unilateral RB, but the median lag time was not significantly different between these two groups; these findings are similar to those in a previous report of patients with RB from Southwest China [4]. There was also no difference in the sex distribution between patients with unilateral and bilateral RB. However, there was a significant difference in treatment modalities between the two groups.

The overall rates of enucleation (15.82%) and mortality (0.90%) were markedly lower in the present study than in other reports of patients with RB in China (> 40%) and other developing counties [4, 9–12]. We speculate that the most likely reason is that the follow-up time was too short (0.5–21.0 months). In the clinical setting, RB can be divided into four development periods according to the tumor development process: the intraocular, glaucoma, extraocular, and systemic metastasis phases. Intraocular RB also can be divided into class A, B, C, D, and E according to the IIRC [13]. The development, prognosis, and outcome of RB require prolonged observation and follow-up. Additionally, a small number of patients were included in this study, possibly affecting the statistical analysis. This may also be a major reason why the heritability (0.46%) was lower than previously reported (25%–35%) [14] and the treatment modalities of the patients over the 3-year study period did not show a reasonable trend. With progression in science and medicine and increased awareness, the rate of conservative treatment should increase and the enucleation rate should decrease on a consistent annual basis. In terms of the risk factors for RB, an incorrect initial working diagnosis or referral diagnosis was associated with a high enucleation rate in our study. However, there was no significant correlation between the lag time and enucleation rate.

Our study has several limitations. First, it was a hospital-based study with not enough number of patients, potentially deviating the results. Second, because of the weakness of most retrospective studies, some important data were not complete, especially the IIRC class, patient ethnicity, and stage of RB at presentation. Thus, many meaningful parameters could not be counted and compared. Third, the follow-up time in our study cohort was relatively short; these patients might experience changes in laterality, treatment, prognosis, second malignancies, survival, and death in the future.

In conclusion, we have herein reported the results of a comprehensive retrospective investigation of 436 patients with RB in China. To the best of our knowledge, this number of patients exceeds most retrospective investigations of RB. Our findings have confirmed that early detection, accurate diagnosis, and active intervention are conducive to control of the disease and retention of patients’ vision. China is still lagging behind many developed countries in terms of prevention and treatment of RB. Neonatal fundus examinations; education for parents and ophthalmologists regarding the early signs of RB, such as leukocoria; and optimization of the therapeutic strategy of RB may improve the outcomes of RB in China and other developing countries.

## MATERIALS AND METHODS

The retrospective observational investigation included all patients with a diagnosis of RB who were examined and managed by the Ophthalmology Department of Quanzhou Maternal and Child Health Hospital (Fujian province, China) from January 2013 to October 2016. The diagnosis was based on clinical examination or pathological findings.

The data collected included age, sex, residence, presenting signs, age at onset, age at diagnosis, lag time (time interval between age at onset and age at diagnosis), family history, medical history, allergy history, physical examination findings, related ocular symptoms, tumor laterality, treatment modalities, and final outcomes. The clinical features at our initial examination included data on visual acuity, intraocular pressure, and the cornea, iris, pupil, lens, vitreous body, and anterior chamber. The referring diagnosis and previous treatments performed elsewhere were also recorded.

All collected data were recorded in an Excel spreadsheet (Microsoft, Redmond, USA) and analyzed using IBM SPSS Statistics 21 (IBM Corp., Armonk, NY, USA) and GraphPad Prism 5 (GraphPad Inc., La Jolla, CA, USA). The Mann–Whitney *U* test was used for unpaired quantitative data, and the chi-square test was used for qualitative data. A *p*-value of < 0.05 was considered statistically significant. This study was approved by the Ethics Committee of Quanzhou Maternal and Child Health Hospital.

## Abbreviations

IIRC: International Intraocular Retinoblastoma Classification
PPV: pars plana vitrectomy
RB: retinoblastoma
